# Histoplasmosis peritonitis in an immunocompetent patient: case report

**DOI:** 10.1186/s12879-024-09083-9

**Published:** 2024-02-14

**Authors:** Thomas Jaugey, Michael Schneider, Cristina Bellini, Stephane Yerly, Charalampos Sotiriadis, Edgardo Pezzetta

**Affiliations:** 1 Department of Surgery, Riviera-Chablais Hospital, Rennaz, Vaud Switzerland; 2 Service of Infectious Diseases, Central Institute, Valais Hospitals, Sion, Switzerland; 3 Histocytopathology, Service of Histocytopathology, Central Institute, Valais Romand Hospitals, Sion, Valais Switzerland; 4 Department of Medical Imaging, Riviera-Chablais Hospital, Rennaz, Vaud Switzerland; 5 Department of Visceral Surgery, Lausanne University Hospital CHUV, Rue du Bugnon 46, Lausanne, Lausanne, 1011 Switzerland

**Keywords:** Disseminated histoplasmosis, Immunocompetent, Abdominal pain

## Abstract

Histoplasmosis is a fungal infection most frequently seen in immunocompromised patients. It is endemic in Central and South America and in Africa. The infection is usually asymptomatic in a healthy individual. Extrapulmonary dissemination can be seen in immunocompromised hosts. Gastrointestinal manifestations frequently involve the terminal ileum and cecum, mimicking Crohn’s disease or malignancy. We describe the case of a 36-year-old healthy man from Cameroon, living in Switzerland for 13 years and without any medical nor surgical history, who presented peritonitis not responding to antibiotics. CT-scan showed bowel obstruction and signs of peritonitis. We opted for an explorative laparoscopy, which was converted to laparotomy with extensive adhesiolysis. Diagnostic of histoplasmosis was confirmed by histology and PCR analysis on biopsy. To our knowledge, this is the first described case of peritonitis as main outcome of a disseminated histoplasmosis involving the peritoneum in an immunocompetent patient.

## Background

Histoplasmosis is a fungal infection most frequently seen in immunocompromised patients or at extreme ages (weaker immune systems). It is endemic in areas such as Central and South America and is present around Mississippi river and in the Ohio River Valley region. It is also present in Sub-Saharan countries [1, 2]. It is transmitted by inhalation of spores of the fungus *Histoplasma capsulatum* that are present in birds or bats feces. In a healthy individual, the infection is usually asymptomatic or can cause flu-like syndrome [3]. In immunocompromised hosts it can rapidly spread to the lung leading to respiratory failure or extrapulmonary dissemination. This form is called disseminated histoplasmosis. Gastrointestinal manifestations are less frequent and involve mostly the terminal ileum and cecum, mimicking Crohn’s disease or malignancy. The treatment is based on Itraconazole for cases of mild to moderate severity and Amphotericin B for the most severe cases. This case report concerns an immunocompetent patient with peritoneal involvement.

## Case history

**Fig. 1:**
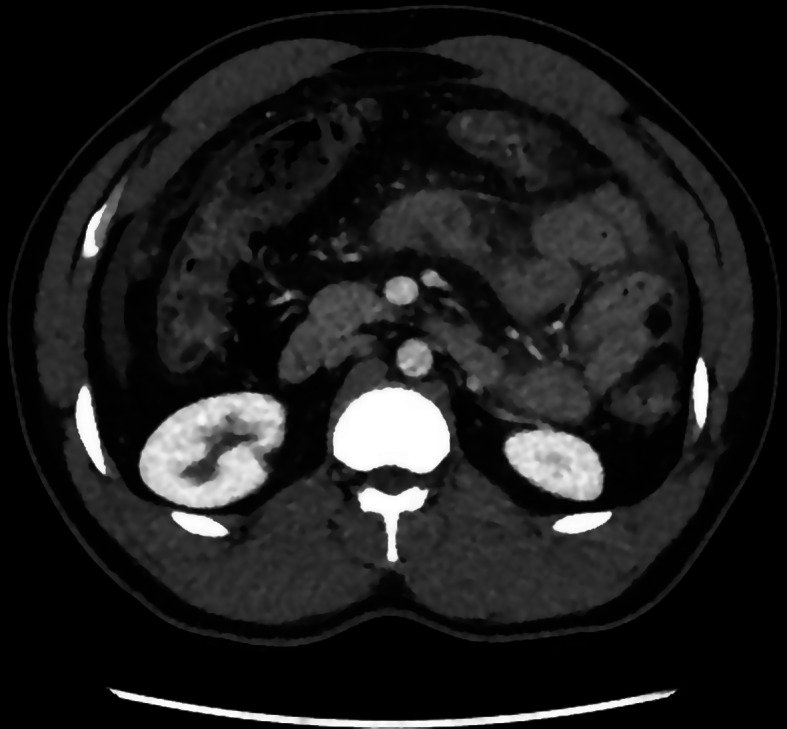
First CT scan with free fluid, reactive mesenteric lymph nodes, peritoneal thickening, mesenteric fat infiltration, wall thickening of the colon and segmental wall thickening of the jejunum

A healthy 36-year-old man from Cameroun living in Switzerland for 13 years and without any medical nor surgical history, suffered of a worsening abdominal pain with bloating, vomiting and feeling feverish. Patient was immunocompetent, has no diabetes and is non-smoker. An abdominal CT-scan (Fig. 1) was performed showing infiltration of mesenteric fat and reactive mesenteric nodes with free abdominal fluid. A left-sided colitis was diagnosed, and the patient received a week of oral Amoxicillin/ clavulanic acid 1 g TID. A colonoscopy was scheduled 6 weeks later. Due to worsening of pain associated to perspiration, the patient presented himself to our emergency department 2 days later.

He described periumbilical pain with radiation to the left iliac fossa. It was the first time he had experienced this kind of pain. He described abdominal discomfort for a few months without diarrhea or weight loss. He worked as a scaffolding aid and did not leave Europe since he last visited Cameroun 13 years ago.

On examination, the abdomen was distended and painful to left abdominal palpation. He didn’t have fever and vital parameters were normal.

The blood test showed a CRP at 134 mg/L (N: <5) without leukocytosis (N: 4–10 G/L) and a slight alteration of liver function tests (ALAT at 88 U/L (N: <50) and gammaGT at 221 U/L) (N: 60)).

**Fig. 2:**
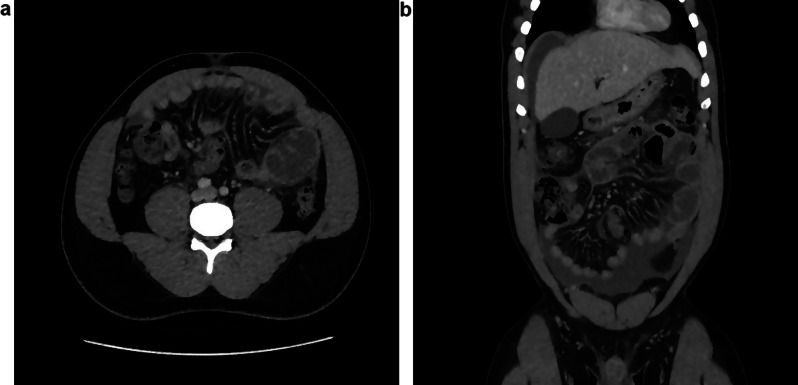
(**a**) and (**b**): CT scan showing jejunal distension, increased intra-abdominal effusion with sign of peritonitis

A new abdominal CT-scan (Fig. 2a and b) found a paralytic ileus picture with jejunal dilation and increased intra-abdominal effusion with signs of peritonitis. The peritoneum seemed to form a shell around all the intestinal loops. It also showed a pancolonic diverticulosis, diverticulitis could not be excluded. The patient was hospitalised for intravenous antibiotic, Ceftriaxone 2 g OD and Metronidazole 500 mg TID. Stool cultures including parasite screening were negative. Bloodwork follow-up showed an improvement of the inflammatory parameters. Given the clinical improvement, he was discharged with an oral treatment of ciprofloxacin 500 mg BID and metronidazole 500 mg TID for a total of 10 days.

**Fig. 3:**
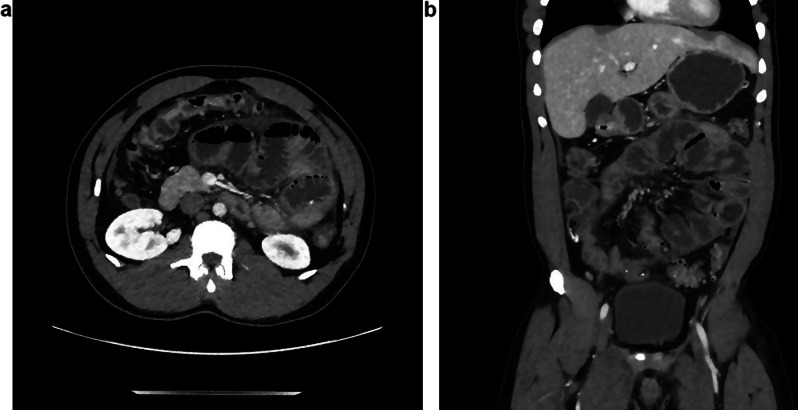
(**a**) and (**b**): CT scan 4 weeks later, showing increased intestinal loop distension but less free fluid

Four weeks after the hospitalisation, he came back to the emergency with recurrence of strong abdominal pain and left guarding. He still had no fever, and vital signs were stable. Blood tests showed no inflammation and liver tests showed only a slight elevated gammaGT (leukocytes 5.2 G/L, CRP 3.9 mg/L, GGT 93 U/L). A new CT scan was performed (Fig. 3a and b), showing a worsening of the ileus and slightly less free fluid compared to the previous CT scan. Additional analyses were done, including Interferon-Gamma Release Assays for tuberculosis infection, stool cultures for *Salmonella, Shigella, Campylobacter*, and 3 search of parasites, that were all negative. The punction of free intraabdominal fluid was cultivated, which came back negative for bacteria, fungi, and mycobacteria. Unfortunately, no cell counts, nor biochemical analysis were performed. As the patient’s status was worrisome and not improving, a laparoscopic exploration was scheduled.

## Material and surgical technique

**Fig. 4:**
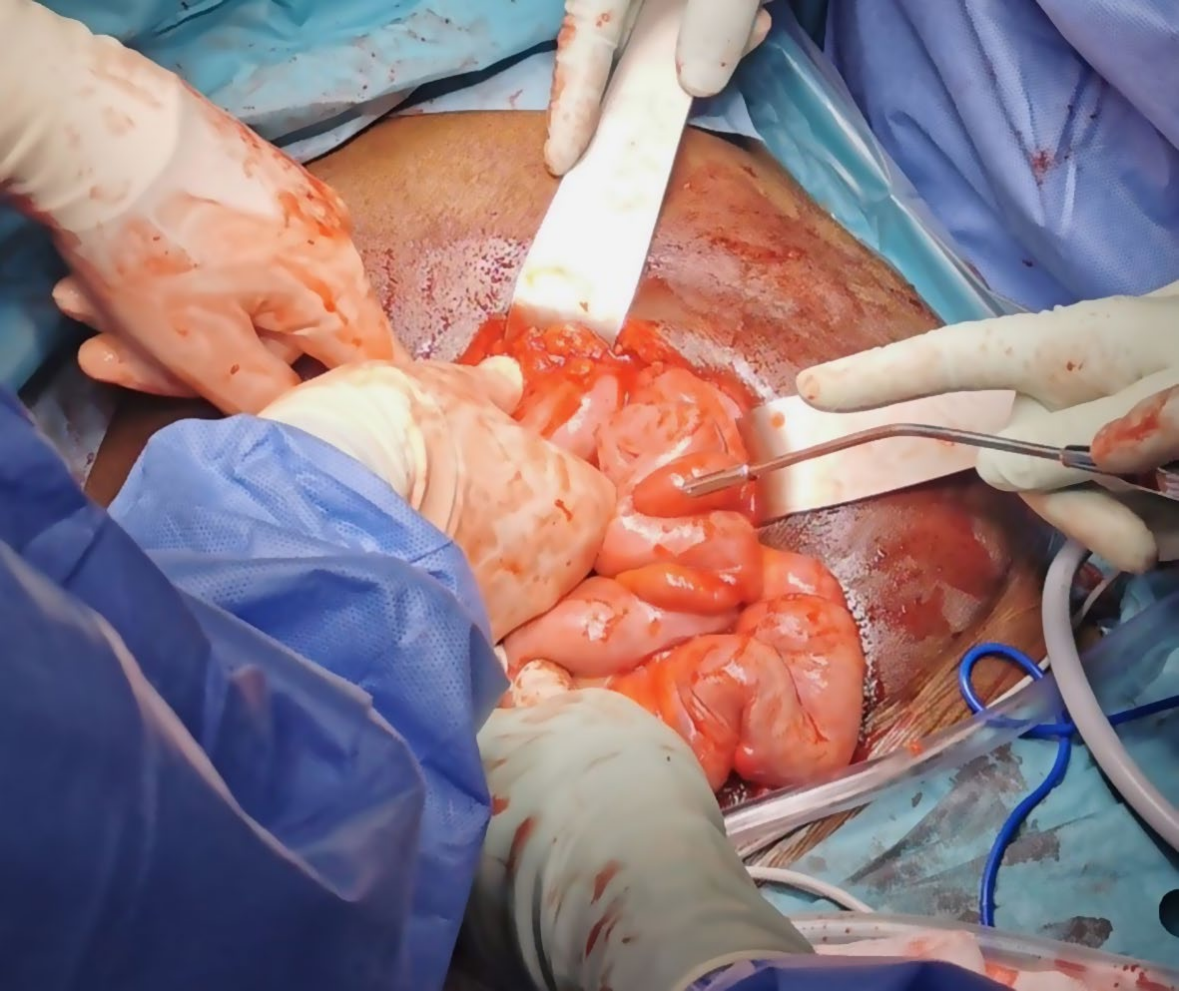
Intraoperative status with thick peritoneum forming a shell around the intestine loops

Laparoscopic exploration was performed in the supine position and under general anaesthesia. We first opted for a laparoscopic approach due to a significant adhesion status, a midline laparotomy was performed. Bloody serous fluid was collected for microbiological cultures and peritoneal biopsies were sent for anatomo-pathologic examination. The entire small intestine was surrounded by a thick fibrous membrane, making the loops inseparable from each other (Fig. 4). Progressive and complete release of the small intestine from the last ileal loop to the angle of Treitz was achieved. The peritoneum was thickened and nodular in places. These nodules were excised. There were no signs of perforation or necrosis, and no abscesses or collections were found.

### Pathological findings

**Fig. 5:**
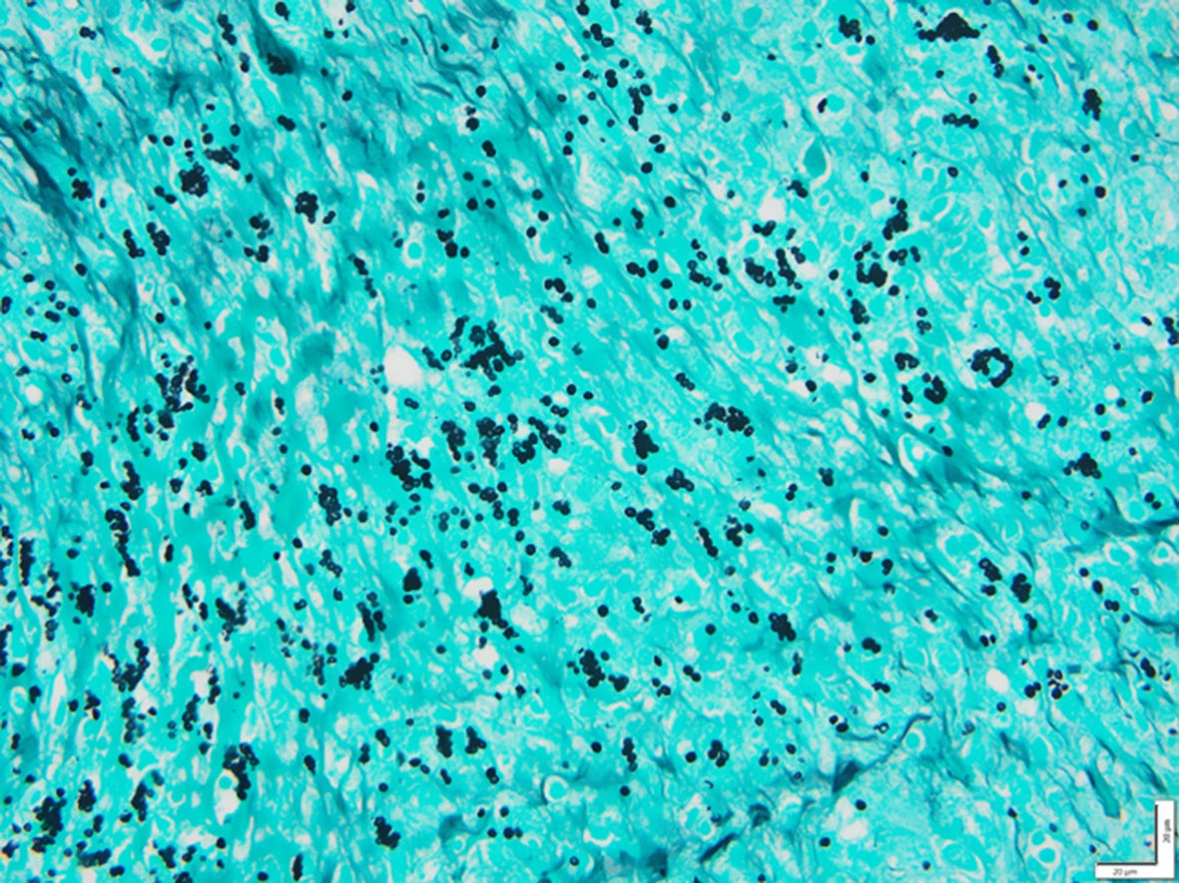
Presence of numerous structures appearing as small spherical or ovoid yeasts compatible with Histoplamsa capsulatum (Grocott Stains, 400X)

The histopathological examination revealed inflammatory large, ovoid granulomatous lesions with few giant cells and central necrosis. On Grocott’s stain (Fig. 5), numerous spore-like mycelial elements with morphology suggestive of *Histoplasma capsulatum*. As the piece was not directly sent for microbiology culture, real-time Polymerase Chain Reaction assay (PCR) of fungal DNA (18 S) on the paraffin block was secondary performed. Results can not differentiate between *Histoplasma capsulatum* or *Blastomyces* sp, but serology studies showed positive serum antibodies against *Histoplasma capsulatum* (immunodiffusion). Urine *Histoplasma capsulatum* antigen (ELISA) was negative. Considering the histopathological finding and the positive serum antibodies assay against *Histoplasma capsulatum*, the diagnosis of histoplasmosis was retained. A chest CT-scan excluded mediastinal lymph nodes involvement or intra-parenchymal lung lesions. By reviewing the abdominal CT scan, we did not find any hepatic or splenic involvement. The patient only presented peritoneal involvement. HIV serology was negative, and CD4 lymphocytes were within normal range (674 G/l, 45%). Protein electrophoresis was also within the normal range.

### Postoperative course and follow-up

A nasogastric tube was maintained until post-operative day (POD) 3 and refeeding began at POD 4. Progressive food intake was well tolerated, and the patient was discharged at POD 8 without any post-operative complication. Symptoms were partially relieved after surgery.

Due to the relatively good health of the patient, the extrapulmonary localization of the fungal infection and the long duration of symptoms (more than one month), antifungal therapy with oral itraconazole was started (200 mg BID). Further evolution was favorable: tolerance to treatment was good, abdominal pain decreased and completely resolved upon 10 months of therapy. Itraconazole blood level was monitored and stayed in steady state. As both serum antibody and urinary antigen control at 12 months were negative, the antifungal therapy could be discontinued. One month after treatment withdrawal, the patient remaind asymptomatic and remains so at one year follow up. Urine antigen and serum antibodies remain negative. We planned a clinical and serological monitoring for at least 12 months after the end of treatment, according to guidelines [4].

## Discussion

Histoplasmosis in immunocompetent patients involving digestive tract is rare. It may present as an asymptomatic infection, a localized infection or can be part of a disseminated disease. Y. Dang et al. [5] found 9 immunocompetent patients with DH and digestive involvement in their literature review. Seven of them touching the colon including 4 of them the terminal ileum. All but one needed antifungal treatment. One patient required thoracotomy for oesophageal involvement secondary to mediastinal histoplasmosis [6]. In addition, we found only 4 other reported cases, which are summarized in Table 1. DH in immunocompetent patients is probably largely misdiagnosed and therefore underreported for several reasons. Most of the patients may be asymptomatic or mildly symptomatic, presenting only transient flu-like symptoms [3]. Usually, symptoms of DH with gastrointestinal involvement are nonspecific (fever, diarrhoea, diffuse abdominal pain). It may mimic inflammatory bowel disease as it usually involves the terminal ileum [7]. In addition, awareness of this infection and low access to diagnostic tests outside but also in endemic areas probably partly explain the underdiagnosis of this disease [2]. Interestingly, Bassey E. Ekeng et al. conducted a descriptive review of cases of gastrointestinal histoplasmosis from 2001 to 2021 and found 212 cases. They found that the clinical presentation was non-specific and differed only slightly between the HIV and non-HIV groups in terms of abdominal pain, which was significantly more frequent in HIV patients [8]. Patients with acute abdomen were described in only 3.1% of cases but without finding peritonitis.

**Table 1:** Review of literature from 2019 to august 2022

Article	Country	Gender	Age	Involvement	Treatment	Prognosis
Dang et al. 2021	China	Male	44	Esophagus + colon	Amp B and Itraconazole	Recovery
Shojaei et al. 2021	Canada	Male	78	Colon	Amp and Itraconazole	Recovery
Nawaz et al.2020	USA	Male	60	Ileocecal	Surgery and Amp B	Recovery
Acharyya et al. 2021	India	Male	8	Ileum	Amp and Itraconazole	Recovery

Amp B = Amphotericin B

Amphotericin B and azoles, like itraconazole, are the 2 main classes of antifungal drugs used in the treatment of histoplasmosis [4]. Amphotericin B eradicated fungemia faster than itraconazole, as serum levels reach steady state faster [9], but toxicity is higher. Therefore, it is mainly used for patients with severe disease including unstable patients. The liposomal preparation of amphotericin B is preferred to the deoxycholate for its comparable efficacy and fewer adverse effects (i.e. nephrotoxicity) [1]. Oral switch to itraconazole can be performed after clinical improvement. Itraconazole alone can be administered for mild to moderate forms. Duration of therapy depend on clinical manifestation, usually for at least 12 months [6, 10]. The need of surgery is rare.

In the literature, we found two cases of patients having bowel obstruction requiring surgical intervention. The patient described by Nawaz et al [11], was on immunosuppressants methotrexate and adalimumab and presented bowel distention due to intestinal obstruction by an intraluminal mass caused by histoplasmosis, requiring ileocecal resection with end ileostomy.

Badyal and al [12] describe a case of bowel distension and peritonitis following perforating colitis. It also led to surgery, with right hemicolectomy and end ileostomy.

Our patient had unexplained peritonitis with unclear and progressive bowel dysfunction The surgery was performed primarily for diagnostic purposes. During the operation, we found significant inflammation of the peritoneum with scattered nodules, without any intestinal obstruction. It required extensive adhesiolysis with peritoneal biopsies which established the diagnosis.

To our knowledge, this is the first described case of an immunocompetent patient presenting histoplasmosis with peritonitis as unique manifestation. Because of the symptoms, neither oesophago-gastroduodenoscopy nor colonoscopy could be performed. We cannot exclude that these examinations could have found intraluminal involvement and possibly avoided surgery. We believe that our patient has contracted the infection during his younger age in Cameroon and that it progressed slowly over the years. We have not found any underlying immunosuppressive condition that could have facilitate the disease.

## Conclusion

We described the case of an immunocompetent patient with peritonitis as the only manifestation of histoplasmosis. Digestive involvement of histoplasmosis in immunocompetent patient is rare and likely underdiagnosed. Gastrointestinal involvement is uncommon and mostly affects the colon or the terminal ileum, mimicking other medical conditions. Clinicians should be aware of this condition, especially for patients originating from or having travelled to endemic countries and not only for immunocompromised patients.

Long term antifungal treatment is necessary for all patients having disseminated histoplasmosis. For responders, serology monitoring should continue for one to two years after treatment withdrawal to avoid relapse.

## Data Availability

All data generated or analysed during this study are included in this published article.
